# Supporting COVID-19 Vaccine Rollout before Charter Class Arrives: The University of California, Irvine Experience

**DOI:** 10.3390/pharmacy9040164

**Published:** 2021-10-09

**Authors:** Alexandre Chan, Melanie D. Joe, Jan D. Hirsch

**Affiliations:** 1 Department of Clinical Pharmacy Practice, School of Pharmacy & Pharmaceutical Sciences, University of California, Irvine, CA 92697, USA; mdjoe@hs.uci.edu (M.D.J.); jdhirsch@uci.edu (J.D.H.); 2 Department of Pharmacy, University of California Irvine Health, Irvine, CA 92697, USA

**Keywords:** vaccination, vaccination clinic, COVID-19, pharmacist

## Abstract

Despite numerous challenges in relation to being a recently established school, the University of California, Irvine (UCI) School of Pharmacy and Pharmaceutical Sciences (SPPS), similar to many schools of pharmacy in the United States, was highly committed to supporting the rollout of COVID-19 vaccines. UCI SPPS and our affiliated UCI Medical Center (UCIMC) Pharmacy Department partnered to spearhead the pharmacy element of a large-scale COVID-19 vaccination clinic on campus for both employees and the community. Three key initiatives were established in order to overcome the obstacles we faced in the large-scale roll out of COVID-19 vaccines: (1) forging new collaborations within the pharmacy team, (2) leveraging interprofessional education and practice, and (3) raising awareness of the pharmacists’ role. Our response to the COVID-19 vaccines at UCI was a tangible, visible model that demonstrated that, while we continue to embrace our role in team-based, patient-centered care, it is also important for us to step up and lead the profession. Additionally, this vaccine rollout experience is a teachable moment for our communities and our health professional partners as we continue to march forward as one voice to serve the American public.

## 1. Introduction

Since late December 2020, our nation has been racing against time to roll out coronavirus disease 2019 (COVID-19) vaccines to the community. Similar to many schools of pharmacy in the United States, the University of California, Irvine (UCI) School of Pharmacy and Pharmaceutical Sciences (SPPS) was highly committed to supporting the rollout of COVID-19 vaccines. However, as a recently established school, we had a limited number of faculty, and as important, the urgency of the rollout of vaccines necessitated that we began before we enrolled our first cohort of pharmacy students, who have proven to be pivotal to the success of COVID-19 vaccination clinics at other schools of pharmacy [1,2]. Furthermore, our newly founded school lacked any appreciable precedent for collaborating with our affiliated UCI Medical Center (UCIMC) and the surrounding community. 

Despite these challenges, the SPPS and UCIMC Pharmacy Department partnered to spearhead the pharmacy element of a COVID-19 vaccination clinic on campus for the community and employees. In the end, between 15th January 2021 and 12th June 2021, over 76,000 vaccine doses were prepared and administered to over 45,000 people. Cascades of teachable moments, often building on knowledge just recently gained from another, occurred during the course of three key initiatives established to overcome the obstacles we faced to support the large-scale roll out of COVID-19 vaccines during such a pressing time.

## 2. Key Initiatives

### 2.1. Initiative 1: Forging New Collaborations within Pharmacy Team

Marshalling our peers (pharmacists and pharmacy technicians) from many practice settings to collaborate was essential to creating a sustainable yet short term team to prepare and administer vaccines. To start, both SPPS and UCIMC Pharmacy Department staff, almost all previously unknown to each other, worked closely together to emulate the successful vaccine preparation and administration services previously established at the UCIMC for the campus-based vaccine clinic. SPPS performed additional outreach to recruit volunteers from a developing network of affiliated pharmacists and alumni network (Anteaters in Pharmacy) to help at the clinic. Since SPPS had not yet enrolled pharmacy students, we worked with the experiential education offices at the other two University of California schools of pharmacy (San Diego and San Francisco) to include over 30 student pharmacists administering vaccines under SPPS faculty and UCIMC pharmacist/pharmacy residents’ supervision. 

### 2.2. Initiative 2: Leveraging Interprofessional Education and Practice

To ensure a full workforce to rollout the vaccines, we organized interprofessional education activities with nursing and medical faculty, who were also largely unknown to each other prior to the clinic. Physicians, nurses, and nursing students were formally trained on-site by pharmacists and technicians with teach-back verification and continuous oversight of aseptic technique. Additionally, we trained over 35 medical and nursing students to prepare vaccines through interprofessional education sessions, providing vaccine-specific instructions and hands-on application and validation of technique. Through these interprofessional activities, we introduced team-based care involving pharmacy as a new player on the UCI campus and effectively established a model of interprofessional education for incoming pharmacy students in the fall of 2021. Notably, interprofessional practice amongst medicine, nursing, and pharmacy was the default mode in the clinic despite there being no initially established structures and processes in the setting. Pharmacists administering vaccines alongside nurses in addition to preparing vaccines was an element of interprofessional practice that evolved very soon after the clinic opened. Shortly afterward, the nurses and physicians who had been trained by pharmacists and technicians began helping to prepare vaccines to accommodate the ever-increasing patient volume. The team players worked out their roles and interactions as they went along to achieve the common and urgent goal of serving patients.

### 2.3. Initiative 3: Raising Awareness of Pharmacists’ Role

The essential role of the pharmacist was on obvious display at the campus vaccine clinic to health professionals, students, community members, and our own UCI employees. Pharmacists and technicians preparing vaccines were not hidden away in a back room of the venue. Instead, all vaccine preparation activity was clearly visible, though secure, as a distinct station of the clinic with overhead signage indicating the new SPPS at work to serve all 45,000 people who came to the clinic. Pharmacists were also highly visible when performing direct patient care activities such as administering vaccines and answering patient questions while staffing vaccine administration tables as well as checking on patients in the after-vaccine observation area. The clinic provided an ideal demonstration of the pharmacist as an expert in all aspects of vaccine acquisition, storage, handling, preparation, and administration and in evolving the clinical patient care team base. 

Beyond the clinic, we also presented two public webinars attended by 400 community members [3]. The goal was to educate and reassure the general public about the COVID-19 vaccines by disseminating timely, easily understood information on the fundamentals and quickly evolving evidence surrounding the COVID-19 vaccines. Drawing on expertise from both departments of the new school (pharmaceutical sciences and clinical pharmacy practice), for the first time, the webinars included public explanations of the scientific basis of the COVID vaccines from a pharmaceutical sciences faculty member as well as information regarding the clinical trials, delivery, and administration issues from two clinical pharmacy practice faculties. 

## 3. Concluding Remarks

We are not alone in our vaccine rollout experience here at UCI. Additionally, we understand that each pharmacist and pharmacy organization had their own series of teachable moments that they and their partners learned from as the COVID-19 testing and vaccines rolled out. As a profession, we have long acknowledged our role in team-based, patient-centered care as well as the lack of societal awareness of our essential role. Our response to the COVID-19 vaccines was a tangible, visible model that demonstrated that, while we continue to embrace these ideas (talk the talk), it is also important for us to step up and lead the profession (walk the walk) and to not be easily derailed by any real or perceived barriers during this pandemic. This is a teachable moment for our communities and our health professional partners as we continue to march forward as one voice to serve the American public. 
